# Tau co-organizes dynamic microtubule and actin networks

**DOI:** 10.1038/srep09964

**Published:** 2015-05-05

**Authors:** Auréliane Elie, Elea Prezel, Christophe Guérin, Eric Denarier, Sacnicte Ramirez-Rios, Laurence Serre, Annie Andrieux, Anne Fourest-Lieuvin, Laurent Blanchoin, Isabelle Arnal

**Affiliations:** 1 Inserm, U836, BP170, 38042 Grenoble, Cedex 9, France; 2 Université Grenoble Alpes, Grenoble Institut des Neurosciences, BP170, 38042 Grenoble, Cedex 9, France; 3 Institut de Recherches en Technologies et Sciences pour le Vivant, iRTSV, Laboratoire de Physiologie Cellulaire et Végétale, CNRS/CEA/INRA/UJF, 38054 Grenoble, France; 4 iRTSV, GPC, CEA, 38054 Grenoble, France

## Abstract

The crosstalk between microtubules and actin is essential for cellular functions. However, mechanisms underlying the microtubule-actin organization by cross-linkers remain largely unexplored. Here, we report that tau, a neuronal microtubule-associated protein, binds to microtubules and actin simultaneously, promoting *in vitro* co-organization and coupled growth of both networks. By developing an original assay to visualize concomitant microtubule and actin assembly, we show that tau can induce guided polymerization of actin filaments along microtubule tracks and growth of single microtubules along actin filament bundles. Importantly, tau mediates microtubule-actin co-alignment without changing polymer growth properties. Mutagenesis studies further reveal that at least two of the four tau repeated motifs, primarily identified as tubulin-binding sites, are required to connect microtubules and actin. Tau thus represents a molecular linker between microtubule and actin networks, enabling a coordination of the two cytoskeletons that might be essential in various neuronal contexts.

A tight coordination between microtubules and actin is required for many cellular functions, such as cell division, migration and morphogenesis^1^. In neurons, this synergistic activity drives the guided extension of axons during neuronal development, as well as the formation and activity of synapses in mature neurons^2^,^3^. Microtubule/actin interactions involved in these processes include various events like the co-alignment and coordinated assembly of both polymers^1^,^4^,^5^,^6^. To date, several candidates have been identified as linkers between the two cytoskeletal systems; however the molecular mechanisms governing this crosstalk are still poorly explored^1^,^3^.

Tau is a neuronal microtubule-associated protein that promotes microtubule polymerization and bundling^7^,^8^,^9^,^10^,^11^, and is responsible for microtubule stabilization in axons^12^,^13^. Tau is involved in the development and maintenance of the nervous system and the deregulation of its function is associated with Alzheimer’s disease and other neurodegenerative pathologies^14^,^15^. Beyond its microtubule-stabilizing properties, tau is also implicated in the regulation of actin cytoskeleton^16^. Accordingly, several *in vitro* studies have reported the association with actin of the full-length tau^17^,^18^ and tau sub-regions containing the microtubule-binding sites^19^,^20^, although this direct interaction has been controverted^21^. *In vivo*, tau co-localizes with actin in differentiating PC12 cells^22^ and in the post-synaptic compartments of mature neurons^23^. Tau has also been found on dynamic microtubules in the actin-rich growth cone areas of developing neurons^24^,^25^. Furthermore, tau neurotoxicity correlates with alterations of actin organization in animal models of Alzheimer’s disease^17^. Overall, these data support a role for tau as a regulator of microtubules and actin, and suggest that tau is a potential linker between the two cytoskeletons as hypothesized some years ago^1^. In the present study, we investigated the ability of tau to simultaneously interact with actin and tubulin. Using reconstituted cell-free systems, we addressed the basic mechanisms underlying tau-mediated co-organization of dynamic microtubule and actin networks.

## Results

### Tau simultaneously binds to microtubules and actin filaments

**Figure 1:**
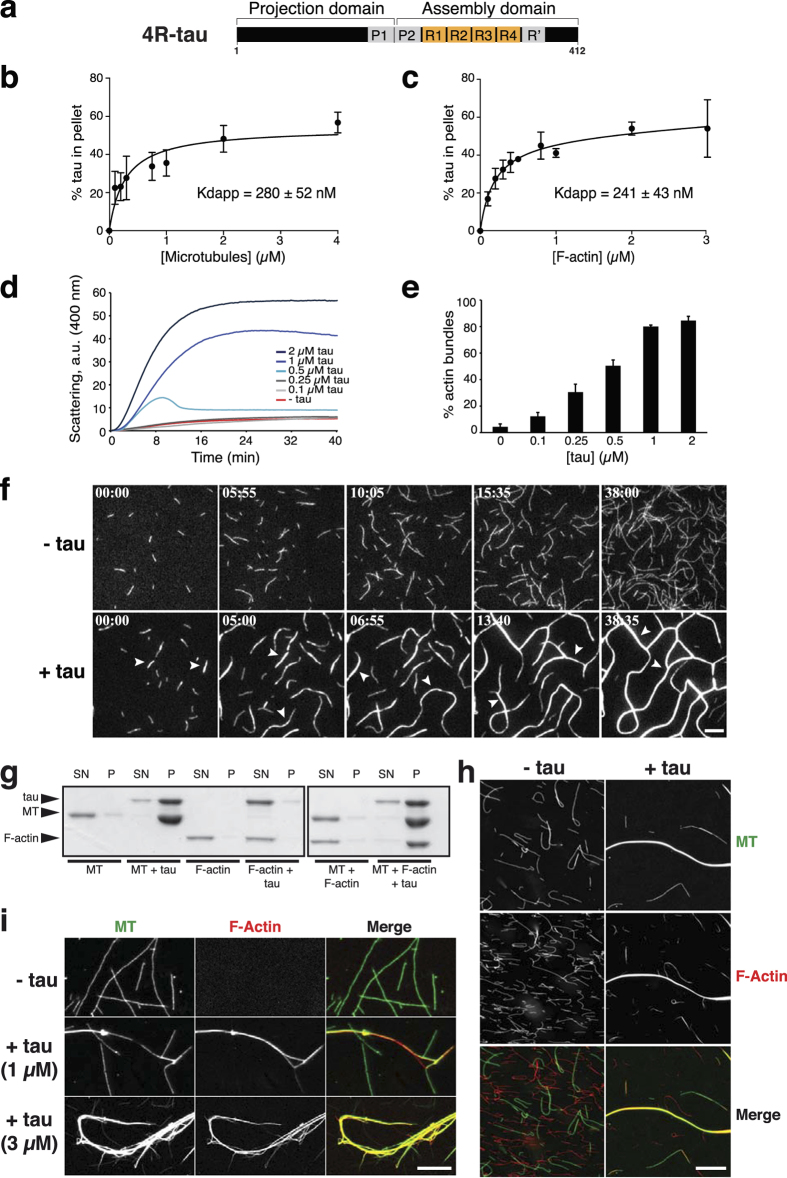
**Tau interacts with microtubules and F-actin, and forms hybrid bundles of both polymers**. (**a**) Scheme of tau isoform used in this study. Tau projection domain contains a proline-rich region (P1). The C-terminal domain consists of a proline-rich region (P2), 4 repeat motifs (R1-R4) and a C-terminal extension comprising a pseudo-repeat motif (R’). (**b**) Tau binding affinity for microtubules. Increasing concentrations of microtubules were incubated with 0.5 μM tau in BRB80-K buffer. Percentage of bound tau was plotted against microtubule concentration and fitted with a hyperbolic function. The Kd_app_ value was 280 ± 52 nM (mean ± SD, five independent experiments). (**c**) Tau binding affinity for F-actin. Increasing concentrations of F-actin were incubated with 0.5 μM tau in AP buffer. Percentage of bound tau was plotted against F-actin concentration and fitted with a hyperbolic function. The Kd_app_ value was 241 ± 43 nM (mean ± SD, three independent experiments). (**d**) Time course of actin bundling (4 μM) with increasing tau concentrations, monitored by light scattering at 400 nm. a.u., arbitrary units. (**e**) Quantification of actin bundles by low speed sedimentation after 1 hour incubation with various tau concentrations. Data represent the mean ± SD (three independent experiments). (**f**) TIRF microscopy of actin (0.4 μM) polymerized with or without 0.4 μM tau. Time 00:00 (min:sec) indicates start of acquisition. Arrowheads, F-actin bundles; scale bar, 10 μm. (**g**) Tau induces microtubules/F-actin complexes. Tau (4 μM) was mixed with either 2 μM taxol-stabilized microtubules, 2 μM phalloidin-stabilized F-actin or both polymers before low-speed centrifugation. F-actin sedimented only with microtubules and tau. P, pellet; SN, supernatant; MT, microtubule. (**h**, **i**) Visualization of tau-induced complexes between phalloidin-stabilized F-actin (red) and taxol-stabilized microtubules (green). (**h**) Microtubules (0.3 μM) and F-actin (0.2 μM) were mixed for 30 minutes with or without tau (0.5 μM) before fixation and observation. Scale bar, 10 μm. (**i**) Microtubules (2 μM) and F-actin (4 μM) were incubated with increasing concentrations of tau before centrifugation at 50,000xg to sediment single/bundled microtubules but not F-actin alone. Scale bar, 10 μm.

Tau can be divided into two large domains: an N-terminal portion, called the projection domain, and a C-terminal part containing three or four repeats of a conserved 31-32 amino-acid stretch (Fig. 1a). These repeat motifs are required for the microtubule-binding and -stabilizing properties of tau^11^,^26^,^27^, and have also been proposed to interact with actin^18^,^19^,^20^. In addition, the regions flanking the repeats (i.e. the proline-rich domain and/or the C-terminal sequence of tau) are implicated in tau interaction with each polymer^11^,^18^,^28^. To study the role of tau in the microtubule/actin interplay, we produced recombinant human tau containing four microtubule-binding sites (4R-tau), which interacted with microtubules (Fig. 1b) and promoted both their assembly and bundling as expected (Supplementary Fig. S1a and b)^7^,^8^,^9^,^10^,^11^. We tested the effect of tau on *in vitro* actin assembly and organization. Our results showed that tau bound to actin filaments (F-actin) with an apparent equilibrium dissociation constant (Kd_app_) of about 241 ± 43 nM (Fig. 1c), a value close to Kd_app_ of tau for microtubules measured by a similar experimental approach (280 ± 52 nM, Fig. 1b). The Kd_app_ value of tau for F-actin was similar in the buffers used either for actin assembly (Fig. 1c) or actin/tubulin co-assembly (Supplementary Fig. S1c). We found that tau did not significantly affect actin assembly properties by using pyrene-actin polymerization and F-actin sedimentation (Supplementary Fig. S1d and e). In contrast, tau influenced actin organization by promoting the apparition of bundled F-actin in a concentration dependent manner, as revealed by light scattering (Fig. 1d) and low-speed sedimentation assays (Fig. 1e, see also Supplementary Fig. S1f). We next visualized, for the first time in real time, the effect of tau on actin organization by Total Internal Reflection Fluorescence (TIRF) microscopy (Fig. 1f, Supplementary Movie S1). In the absence of tau, only single actin filaments could be observed whereas, in the presence of tau, they progressively formed long and thick F-actin bundles. Quantification of actin growth rate at early time points indicated that tau did not significantly changed the elongation speed of actin compared to the control (1.78 ± 0.28 and 1.76 ± 0.41 μm/min, respectively). Overall, our data indicate that tau is an actin-binding and -bundling protein in agreement with previous studies showing the direct interaction of the full-length tau with actin filaments^17^,^18^. These results are consistent with an actin-scaffolding role for tau that has been recently proposed to regulate the morphological changes underlying synaptic plasticity in mature neurons^23^.

The ability of tau to interact with F-actin raises the possibility that tau operates as a cross-linker between microtubules and actin filaments. To investigate this hypothesis, we set up sedimentation conditions to assay tau interaction with pre-polymerized microtubules and actin filaments. Tau was added to taxol-stabilized microtubules and/or phalloidin-stabilized F-actin, and reactions were centrifuged at very low speed on a sucrose cushion (Fig. 1g). In these conditions, microtubules bundled by tau were pelleted, while all other polymers, i.e. single microtubules, single F-actin and tau-induced bundled F-actin, remained in the supernatant (Fig. 1g, left panel). In contrast, F-actin sedimented only when incubated with microtubules and tau (Fig. 1g, right panel), suggesting that tau simultaneously interacts with both cytoskeletons and promotes the formation of large macro-molecular complexes. We next visualized fluorescently-labelled pre-polymerized microtubules and actin filaments with or without tau (Fig. 1h). In the absence of tau, single microtubules and F-actin were randomly distributed in the solution (Fig. 1h, left), whereas hybrid bundles of co-aligned microtubules and actin filaments appeared in the presence of tau (Fig. 1h, right). Using co-sedimentation assays onto coverslips in conditions where F-actin did not sediment unless associated with single or bundled microtubules, we observed mixed arrays of microtubules and F-actin only in the presence of tau (Fig. 1i). This result supports the idea that tau tightly connects the two cytoskeletons.

### Tau coordinates dynamic microtubules and actin filaments

**Figure 2:**
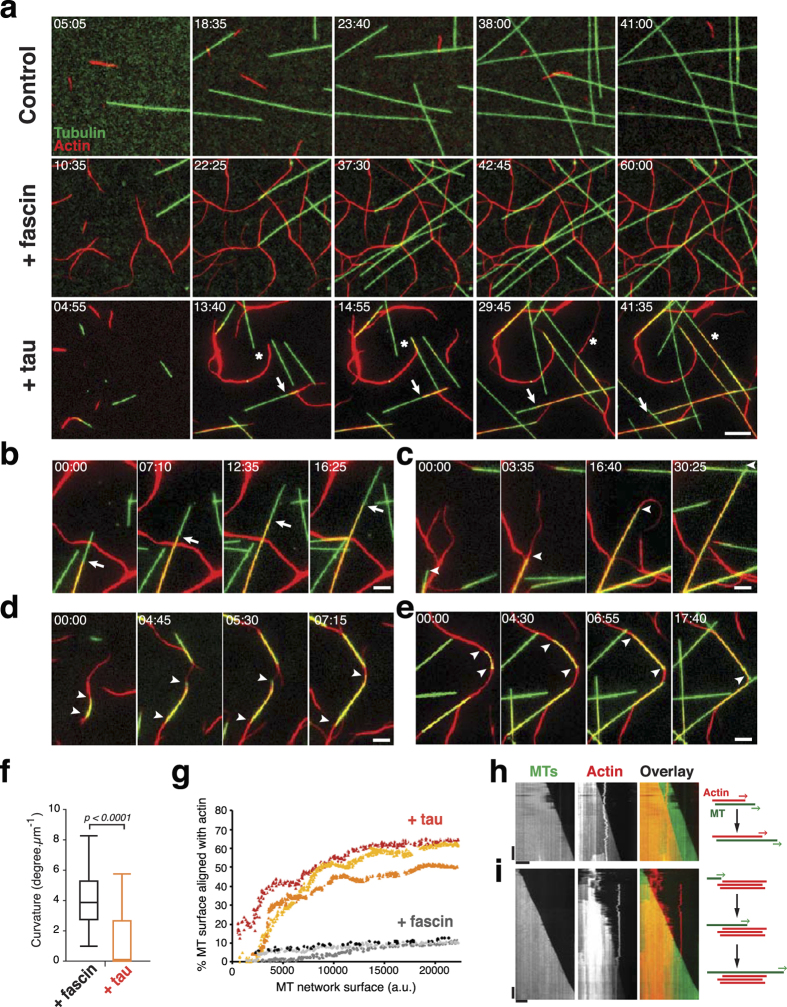
**Tau coordinates dynamic microtubules and actin filaments**. (**a**) TIRF microscopy of co-polymerizing microtubules and F-actin. In the control (upper panel), 20 μM tubulin dimers (green) were co-assembled with 0.4 μM actin monomers (red). Most actin filaments were floating out of the TIRF field due to a lower medium viscosity compared to conditions used for actin assembly (see Methods). Addition of 1 μM fascin (middle panel) induces the apparition of a network of F-actin bundles randomly distributed around microtubules. Tau at 0.7 μM (lower panel) co-organizes growing microtubules and F-actin. In this last condition, we decreased tubulin concentration to 5 μM to avoid extensive microtubule self-assembly induced by tau. White stars show zippering events between microtubules and actin filaments, arrows point actin filaments growing along microtubules. Time 00:00 (min:sec) indicates start of acquisition. Scale bar, 10 μm. (**b**-**e**) Detailed views of typical microtubule/actin coupling events observed in the presence of tau: guided growth of F-actin along the microtubule wall (**b**), straightening of curved actin filaments by a growing extremity of a microtubule (**c**) and single microtubules elongating along actin bundles (**d**, **e**). White arrows and arrowheads indicate extremities of growing F-actin and microtubules, respectively. Time 00:00 (min:sec) indicates start of event. Scale bar, 5 μm. (**f**) Curvature of actin filaments co-assembled with tubulin and either fascin or tau. Boxes and whiskers represent 25–75 and 5–95 percentiles, respectively and lines within boxes indicate median values (3.89 and 0.094 degree.μm^−1^ with fascin and tau, respectively). Data were compared using the Mann-Whitney test (n = 346 and 354 actin filaments for fascin and tau, respectively). Quantification was performed on fixed time-point images displaying similar microtubule densities. (**g**) Percentage of microtubule surface co-aligned with F-actin as a function of microtubule network surface in the presence of tau or fascin (three representative curves). a.u., arbitrary units. (**h**, **i**) Kymographs of co-aligned growing microtubules and F-actin in the presence of tau. Schemes of microtubule/actin co-organization are drawn on the right. Horizontal bars, 10 μm; vertical bars, 3 min.

We next wondered whether tau was able to co-organize microtubules and actin filaments while assembling. For this purpose, we reconstituted a minimal and original system, in which concomitant self-polymerization of both tubulin and actin could be followed by live TIRF microscopy. To our knowledge, this is the first *in vitro* assay allowing the visualization of dynamic tubulin and actin polymers simultaneously. Microtubules and actin filaments were elongated from a mixture of actin monomers (0.4 μM) and tubulin dimers (20 μM) containing small fractions of fluorescently-labelled actin monomers and tubulin dimers. In the absence of tau, single microtubules and actin filaments assembled independently without any significant contact (Fig. 2a, Supplementary Movie S2). Addition of fascin, a specific actin-bundler^29^, induced cross-linking of actin filaments, but neither affected microtubule organization nor promoted microtubule/actin interactions (Fig. 2a, Supplementary Movie S2). In contrast to fascin, tau was able to coordinate microtubule and actin networks by progressively co-aligning growing microtubules and growing actin filaments (Fig. 2a-e, Supplementary Movie S3). Coupling of microtubule and actin assembly in the presence of tau was mostly characterized by the following events: guided polymerization of single or bundled actin filaments along single or bundled microtubule tracks (Fig. 2a,b, Supplementary Movies S3 and S4a) and progressive straightening of curved F-actin by the end of elongating microtubules (Fig. 2c, Supplementary Movie S4b). Accordingly, the curvature of actin filaments significantly decreased compared to that of actin filaments co-polymerized with tubulin and fascin, illustrating the microtubule-dependent deformation of F-actin in the presence of tau (Fig. 2f). We also observed the growth of single microtubules along F-actin cables (Fig. 2c-e, Supplementary Movie S4c) and, in some cases, thick actin filament bundles could shape and deform microtubules elongating along their tracks (Fig. 2d,e, arrowheads, and Supplementary Movie S4c). We next quantified the microtubule/actin crosslinking activity of tau by measuring the percentage of microtubules co-aligned with actin filaments. Results indicate that the proportion of the microtubule network associated with F-actin increases up to 60% as a function of the total microtubule surface, whereas it did not exceed 14% in the presence of fascin (Fig. 2g).

We then asked whether the physical association of microtubules and actin filaments induced by tau affected their own assembly properties. Fig. 2h,i show representative kymographs of co-aligned microtubules and actin filaments: a single actin filament growing along a single microtubule (Fig. 2h) and a microtubule encountering bundled F-actin (Fig. 2i). Measurements of growth rates indicated that the microtubule speed (1.28 ± 0.17 μm/min, n = 52) did not change when microtubule-growing ends encountered actin filaments and co-aligned (1.31 ± 0.16 μm/min, n = 41). The elongation rate of F-actin polymerizing along microtubule tracks (1.21 ± 0.16 μm/min, n = 80) was also very similar to the one measured for isolated actin filaments (1.29 ± 0.29 μm/min, n = 35). Thus, the tight association and coupled growth of microtubules and F-actin did not modify the intrinsic assembly properties of each polymer. Overall, our results reveal that tau promotes the co-alignment of microtubules and actin filaments, which allows the two networks to adopt the same global organization and direction of growth.

### Tau bridges microtubules and actin *via* its tubulin-binding sites

**Figure 3:**
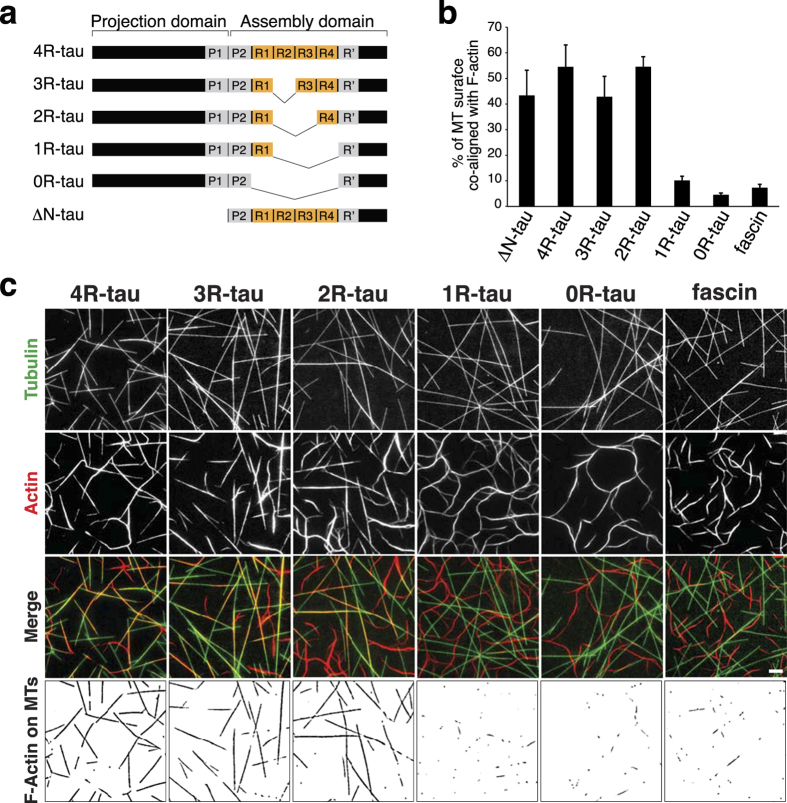
**Tau microtubule-binding sites are required for microtubule/actin crosslinking**. (**a**) Schematic representation of the different tau constructs used in this study. (**b** and **c**) Microtubule/F-actin cross-linking activity of tau proteins. Microtubules and F-actin were co-assembled from tubulin dimers and actin monomers in the presence of the various tau proteins and analyzed by TIRF microscopy (see Methods for details). (**b**) Quantification of the percentage of microtubule surface co-localizing with F-actin from TIRF images at fixed time points exhibiting similar microtubule densities (see examples in (**c**)). Fascin that does not bind to microtubules serves as a negative control. For statistical analysis, we used a non-parametric Kruskal-Wallis ANOVA test followed by post-hoc Dunn comparisons. P values calculated in comparison to the 4R-tau conditions are 0.0626 (1R-tau), 0.0009 (0R-tau) and 0.0286 (Fascin). Error bars represent standard deviations (three to six independent experiments). (**c**) Examples of fixed time point images used to quantify the percentage of microtubule surface co-aligned with F-actin in various conditions. Images were taken at similar microtubule densities. The masked images (lower panel) represent the microtubule area that co-localizes with actin. Scale bar, 10 μm.

**Figure 4:**
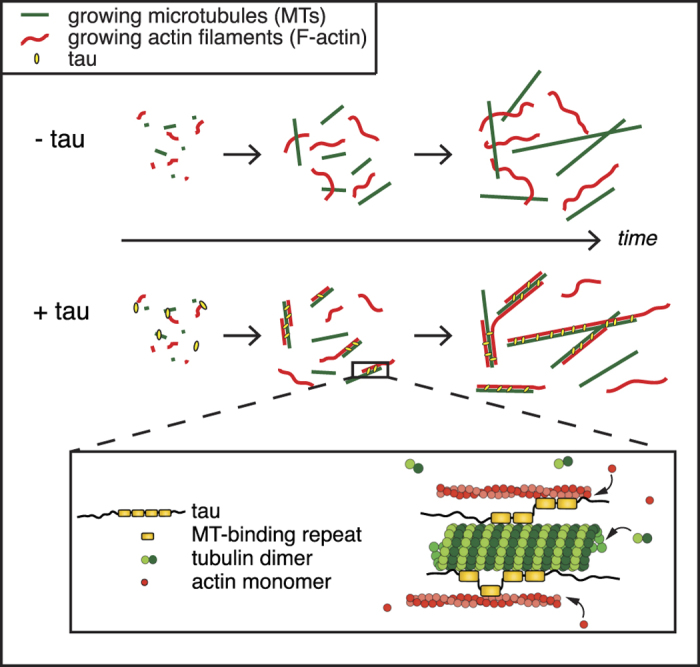
**Model for tau-mediated coordination of microtubule and actin cytoskeletons.** Tau promotes the co-alignment and coupled growth of microtubules (green) and actin filaments (red). We suggest that one tau molecule would bind both actin and microtubules simultaneously and that the distribution of tubulin-binding repeated sites of tau would be required to establish the structural link between the two cytoskeletal polymers (inset). At least two repeats are necessary for the crosslinking activity of tau, with one repeat interacting with microtubules and the other one with actin filaments.

To evaluate which sub-regions of tau serve as bridges between microtubules and F-actin, we generated tau mutants deleted from either the N-terminal projection domain or various numbers of repeats (Fig. 3a and Supplementary Fig. S2a). Indeed, the projection domain might regulate microtubule spacing within bundles possibly *via* homodimerization^9^,^30^, a mechanism that could be critical for bridging microtubules and F-actin. To test this hypothesis, we studied the effect of the N-terminal deleted form of tau (ΔN-tau) on microtubule and actin co-organization. Our results show that ΔN-tau binds to F-actin and to microtubules independently (Supplementary Fig. S2b and c), and cross-links both polymers in growing conditions similarly to the full-length tau (Fig. 3b, Supplementary Movie S5). This suggests that the C-terminal part of tau is sufficient to establish a physical link between microtubules and actin filaments and that tau dimerization through its N-terminal part is unlikely involved in this process. We next produced mutants lacking one to four repeats (3R-, 2R-, 1R- and 0R-tau, Fig. 3a) and examined their ability to co-align dynamic microtubules and actin filaments by TIRF microscopy (Fig. 3c, Supplementary Movie S6). Since the activity of tau to promote microtubule self-assembly decreases with the number of repeat motifs^11^, the concentrations of tau proteins and tubulin were adjusted to ensure that actin polymerization, which was not influenced by tau, and tubulin polymerization started almost simultaneously (Supplementary Movie S6, see also Methods). The different deleted forms of tau still associated independently with microtubules and F-actin in similar concentration ratios of tubulin:tau and actin:tau used for the TIRF assay (Supplementary Fig. S2b and c). To evaluate the influence of tau repeats on microtubule/actin coupling, we measured for each tau mutant the proportion of the microtubule surface co-localizing with F-actin at similar microtubule densities (Fig. 3b,c, bottom panel, Supplementary Movie S6). In the presence of tau proteins with 2, 3 and 4 repeats, about 50% of the microtubule network co-aligned with actin filaments. In contrast, the deletion of more than two repeats (1R- and 0R-tau) almost abolished the link between the two cytoskeletal arrays (Fig. 3b,c). In these conditions, the percentage of the microtubule surface associated with actin filaments strongly decreased to values comparable to those obtained with fascin (Fig. 3b). These data indicate that the repeat motifs are crucial for the microtubule/actin cross-linking activity of tau. Our results suggest a mechanism whereby a minimum of two tau repeats are required to bridge polymers, with one repeat bound to microtubules and the other one to actin filaments (Fig. 4).

## Discussion

In neurons, several molecules have been identified as key players in the microtubule/actin crosstalk, including proteins that simultaneously bind to microtubules and F-actin (e.g. members of the spectraplakin family^31^,^32^) or multi-component complexes of microtubule- and actin-binding factors (e.g. the microtubule end-binding protein EB3 and the actin-binding protein drebrin^33^). The interaction of tau with microtubules in neurons is well documented and more recent studies have proposed that tau also binds to actin^34^. Our work demonstrates that tau acts as a molecular linker between microtubules and actin filaments, enabling the *in vitro* co-organization and coupled growth of the two networks (Fig. 4). These data provide the first evidence for a direct role of tau in the dynamic coordination of microtubule and actin cytoskeletons. We propose a mechanism in which the repeat motifs of tau establish a structural connection between microtubules and actin filaments through the distribution of repeats between the two polymers (Fig. 4). Several phosphorylation sites within the repeats and adjacent sequences have been shown to finely regulate the microtubule-binding and -stabilizing properties of tau^11^,^28^,^35^. Whether and how these phosphorylation sites modulate the affinity of tau for F-actin, and possibly its microtubule/actin cross-linking activity, remain to be elucidated. Interestingly, a recent *in vitro* study has reported the alignment of microtubules along pre-existing actin arrays *via* the cross-linking activity of plus-end tracking proteins at the growing extremity of microtubules^36^, highlighting that the microtubule/actin cross-talk involves different mechanisms based on both lattice and end-binding factors.

The intrinsic capacity of tau to connect the two cytoskeletal systems suggests that it might be at play in the global cytoskeleton reorganization during neuronal development. Tau could contribute to the co-alignment and tandem polymerization of microtubules and actin filaments, the guided growth of microtubules along actin cables, or the microtubule-dependent actin reorganization that have been described in the growth cone of elongating axons and at axon branching points^1^,^4^,^5^,^6^. Along this line, several studies have reported a role for tau in neurite outgrowth, although details about the implication of tau in axonal guidance are unclear^25^,^37^,^38^,^39^. Furthermore, tau has been recently found in the synaptic compartment of mature neurons^23^,^40^ where it may participate to the microtubule/actin coordination that control synapse morphology and functions^2^,^41^,^42^.

Overall, our work advances our understanding of tau’s cytoskeleton-regulating properties and provides new insights into the basic mechanisms that govern the coordination between microtubule and actin cytoskeletons. Given the role of tau in Alzheimer’s disease and related tauopathies, it is essential to elucidate how tau interacts with microtubules and actin in order to decipher molecular pathways leading to cytoskeleton alterations in these pathologies.

## Methods

### DNA constructs and reagents

We used tau isoforms containing three or four repeats (R) at their C-terminus and one N insert at their N-terminus (1N3R- and 1N4R-tau, respectively named 3R- and 4R-tau in the present study). The cDNA of these two isoforms subcloned in the pDEST17 vector were kindly provided by Dr. N. Sergeant (Lille, France). The following tau constructs were generated by a PCR-based strategy (amino-acid numbers are given according to the longest 2N4R-tau isoform): ∆N-tau corresponding to amino acids S198-L441 and repeat-deleted constructs 2R-tau, 1R-tau, 0R-tau in which V275-Q336, V275-K369 and Q244-K369 domains were deleted respectively. When not stated, reagents were from Sigma.

### Protein purification

His-tagged recombinant tau proteins were purified using Talon metal affinity resin (Clontech) and processed by size exclusion in BRB80 buffer (80 mM Pipes, 1 mM EGTA, 1 mM MgCl_2_, pH 6.8). Human fascin and profilin were purified as described^43^,^44^. Tubulin was purified from bovine brain and labelled with ATTO-488^45^ (ATTO-TEC GmbH, Germany). Actin was purified from rabbit skeletal muscle and labelled with Alexa-568 or pyrene^43^.

### Tau interaction with microtubules or F-actin

Taxol-stabilized microtubules were prepared by polymerizing 70 μM tubulin at 36 °C in BRB80 buffer with 1 mM GTP. After 60 min, 70 μM taxol was added and microtubules further incubated 30 min at 36 °C, before being centrifuged for 5 min at 230,000 x *g* and resuspended in BRB80 buffer. 4R-tau (0.5 μM) was incubated with increasing concentrations of taxol-stabilized microtubules for 30 min at room temperature (RT) in BRB80 buffer supplemented with 50 mM KCl (BRB80-K). Samples were centrifuged for 15 min at 230,000 x *g* and analysed by SDS-PAGE. The amount of 4R-tau bound to microtubules in the pellets was quantified by densitometry on Coomassie-stained gels using Image J software (version 1.49 g, NIH, USA). The percentage of bound 4R-tau was plotted versus microtubule concentrations and the apparent equilibrium dissociation constant was calculated from a fitted hyperbolic curve using Prism 6 (GraphPad software, USA). In the absence of microtubules, no tau was detected in the pellet indicating that tau was soluble under these conditions. To determine tau affinity for F-actin, phalloidin-stabilized F-actin was prepared from 20 μM G-actin incubated for 1h at RT with 20 μM phalloidin in AP (Actin Polymerization) buffer containing 2 mM Tris pH 8.0, 0.2 mM ATP, 0.5 mM DTT, 0.1 mM CaCl_2_, 50 mM KCl, 1 mM MgCl_2_, 1 mM EGTA, 10 mM imidazole. Increasing amounts of phalloidin-stabilized F-actin were incubated with 0.5 μM 4R-tau in AP buffer for 30 min at RT. Samples were centrifuged at 100,000 x *g* for 15 min and the amount of 4R-tau bound to F-actin in the pellets was quantified as described above. Mean values of dissociation constants were calculated from the average of at least three independent experiments. In the absence of actin, no tau was detected in the pellet.

### Actin bundling assays

Increasing amounts of 4R-tau were added to 4 μM G-actin and the formation of F-actin bundles was monitored by light scattering at 400 nm. The amount of bundles was quantified after 1 hour incubation by sedimentation as described above with a centrifugation speed of 15,000 x *g* for 10 min to selectively pellet bundled F-actin.

### Cross-linking activity of tau on pre-formed microtubules and F-actin

Various combinations of taxol-stabilized microtubules (2 μM), phalloidin-stabilized F-actin (2 μM) and 4R-tau (4 μM) were incubated in BRB80 buffer for 30 min at RT and centrifuged on a 30% sucrose cushion for 10 min at 4,000 x *g*. Supernatants and pellets were analyzed by SDS-PAGE. To visualize microtubule/F-actin complexes, fluorescent taxol-stabilized microtubules were prepared from tubulin containing 10% of fluorescent ATTO-565 tubulin. Fluorescent taxol-stabilized microtubules (0.3 μM) and fluorescent ATTO-488 phalloidin-stabilized F-actin (0.2 μM) were incubated for 30 min at RT in BRB80 buffer with or without 4R-tau (0.5 μM) and examined under the fluorescence microscope (Zeiss Axioskop). For sedimentation onto coverslips, fluorescent taxol-stabilized microtubules (1 μM), fluorescent phalloidin-stabilized F-actin (2 μM) and increasing amounts of 4R-tau were incubated in BRB80 buffer at RT for 40 min (sample volume of 20 μl). Reactions were stopped by addition of 1 ml of BRB80 buffer containing 0.5% glutaraldehyde and 25% (w/v) sucrose. Samples were loaded onto 7 ml of 10% glycerol cushion, centrifuged onto coverslips for 30 min at 50000 x *g*, fixed in methanol and observed under the fluorescence microscope.

### TIRF microscopy

Perfusion chambers were prepared with functionalized silane-PEG (Creative PEGwork) glass slides, as described previously^46^. For actin polymerization, the flow cell was incubated with PLL-g-PEG (2 kDa, 0.1 mg/mL in 10 mM Hepes, pH 7.4, Jenkem) and washed with 1% BSA in AP-buffer. Actin growth was initiated by flowing 0.4 μM G-actin (containing 30% Alexa-468 labelled G-actin) together with 0.1 μM 4R-tau in AP-buffer containing 0.2 mM ATP, 0.3 μM profilin, 4 mM DTT, 1% BSA, 1 mg/ml glucose, 70 μg/ml catalase, 580 μg/ml glucose oxidase and 0.3% methylcellulose. For actin and tubulin co-assembly, the flow cell was incubated with PLL-g-PEG, then washed with 1% BSA in BRB80 buffer. Microtubule and actin growth was initiated by flowing tubulin dimers (containing 30% ATTO-488 labelled tubulin) and G-actin (containing 30% Alexa-568 G-actin) in the absence or in the presence of tau proteins in BRB80-K buffer containing 1 mM GTP, 4 mM DTT, 1% BSA, 0.4 μM profilin, 1 mg/ml glucose, 70 μg/ml catalase, 580 μg/ml glucose oxidase and 0.2% methylcellulose. These specific buffer conditions allowed simultaneous polymerization of actin and tubulin with a similar nucleation lag time, in the range of the protein concentrations used. Note that the final ionic strength of the buffer is 210 mM (calculated from Thiede *et al.*, 2013^47^), close to physiological one. For this assay, we had to decrease the methylcellulose concentration (0.2%) compared to that used to visualize actin assembly alone (0.3%) in order to avoid unspecific microtubule bundling. At this lower methylcellulose concentration, single actin filaments hardly remained in the TIRF field, unless they were bound to microtubules and/or bundled. The concentration of actin was 0.4 μM in all conditions. In control experiments without tau or in the presence of fascin (1 μM), tubulin was polymerized at 20 μM. In the presence of 4R-tau (0.7 μM), we decreased the tubulin concentration to 5 μM (tubulin:tau ratio of 7:1), in order to avoid extensive microtubule nucleation that occurred upon the addition of tau and rendered the analysis of microtubule/F-actin interactions impossible. The tau constructs differentially affected microtubule self-assembly. In particular, deletion of the repeats reduced the ability of tau to promote tubulin nucleation. The ratios of tubulin to tau proteins were thus held at 7:1 but adjusted as follows: 5 μM tubulin for 0.7 μM ∆N-tau, 15 μM tubulin for 2.1 μM 3R-tau, 20 μM tubulin for 2.8 μM 2R-tau and 21.5 μM tubulin for 3 μM 1R-tau or 0R-tau. This ensured that, for each tau construct, tubulin and actin polymerization roughly started at the same time. Samples were visualized on an inverted microscope (Eclipse Ti, Nikon) equipped with an ilas^2^ TIRF system (Roper Scientific), a cooled charged-coupled device camera (EMCCD Evolve 512, Photometrics) with 512 × 512-pixel imaging array (16 × 16 μm pixels), a warm stage controller (LINKAM MC60), and controlled by MetaMorph software (version 7.7.5, Molecular Devices). Samples were excited with 491- and 561 nm lasers and observed using an Apochromat 60X oil immersion objective (NA 1.49). Dual-color time-lapse imaging (488 and 561 nm) was performed at 32 °C for actin/tubulin co-assembly and 26 °C for actin polymerization, during 45 min at 1 frame per 5 s with a 100 ms exposure time.

### Image analysis

All image analysis was performed using ImageJ^48^. To quantify microtubule/actin overlap, masks corresponding to the actin and microtubule networks were generated with a homemade ImageJ macro and used to measure the surface of microtubules co-localizing with F-actin on each stack image. The ratio of the microtubule surface co-localized with actin to the total surface of the microtubule network was represented as a function of the total microtubule network surface. To compare the microtubule/actin co-localization induced by each tau-deleted forms, the same procedure was applied to images with similar microtubule densities. Results were averaged from at least three independent experiments. The elongation rates of microtubules (either single or associated with F-actin) and of F-actin polymerizing along microtubule tracks were determined on kymographs using ImageJ software and a homemade KymoTool. Because single actin filaments were very motile, their elongation rate was derived from actin length measurements on image stacks. To evaluate F-actin curvature in presence or absence of tau, the masks (generated as explained above) of actin networks were skeletonized. Actin filament segments between crossings were recovered, manually traced and interpolated. The sum of the angles formed by 3 consecutive points (10 pixels apart) along the selected line was calculated and divided by the length of the line. Thus the calculated curvature is as small as the line is straight and long. The procedure was applied to actin networks co-assembled with tubulin in presence of either fascin or tau, and on images exhibiting similar microtubule densities.

### Statistical analysis

All statistical analysis was performed as implemented in Prism 6.0 (GraphPad Software, La Jolla, USA).

## Author Contributions

All authors contributed to the design and the interpretation of experiments. A. E., E. P., C. G., S. R. R., L. S and A. F. L. prepared the reagents, performed the experiments and analysed the data. A.A., L.B. and I.A. analysed the data. A. E. and E. D. performed image analysis. A. E. and I. A. wrote the paper. All authors reviewed the manuscript.

## Additional Information

**How to cite this article**: Elie, A. *et al*. Tau co-organizes dynamic microtubule and actin networks. *Sci. Rep.* **5**, 09964; doi: 10.1038/srep09964 (2015).

## Supplementary Material

Supporting Information Supplementary Movie S1

Supporting Information Supplementary Movie S2

Supporting Information Supplementary Movie S3

Supporting Information Supplementary Movie S4

Supporting Information Supplementary Movie S5

Supporting Information Supplementary Movie S6

Supporting Information Supplementary Figures and Supplementary Movie Legends
