# Supplementary material for: Tau co-organizes dynamic microtubule and actin networks
Source: Sci Rep. 2015 May 5;5:9964. doi: 10.1038/srep09964 (PMC4421749; doi:10.1038/srep09964)
Supplement: Supporting Information — Supplementary Figures and Supplementary Movie Legends [file srep09964-s7.pdf]

Supplementary Information

Tau co-organizes dynamic microtubule and actin networks

Auréliane Elie[1,2], Elea Prezel[1,2], Christophe Guérin[3], Eric Denarier[1,2,4], Sacnicte Ramirez-Rios[1,2], Laurence Serre[1,2], Annie Andrieux[1,2,4], Anne Fourest-Lieuvin[1,2,4], Laurent Blanchoin[3] and Isabelle Arnal[1,2,*]

[1]Inserm, U836, BP170, 38042 Grenoble, Cedex 9, France

[2]Université Grenoble Alpes, Grenoble Institut des Neurosciences, BP170, 38042 Grenoble, Cedex 9, France

[3]Institut de Recherches en Technologies et Sciences pour le Vivant, iRTSV, Laboratoire de Physiologie Cellulaire et Végétale, CNRS/CEA/INRA/UJF, 38054 Grenoble, France

[4]iRTSV, GPC, CEA, 38054 Grenoble, France

[*]Corresponding author: isabelle.arnal@ujf-grenoble.fr

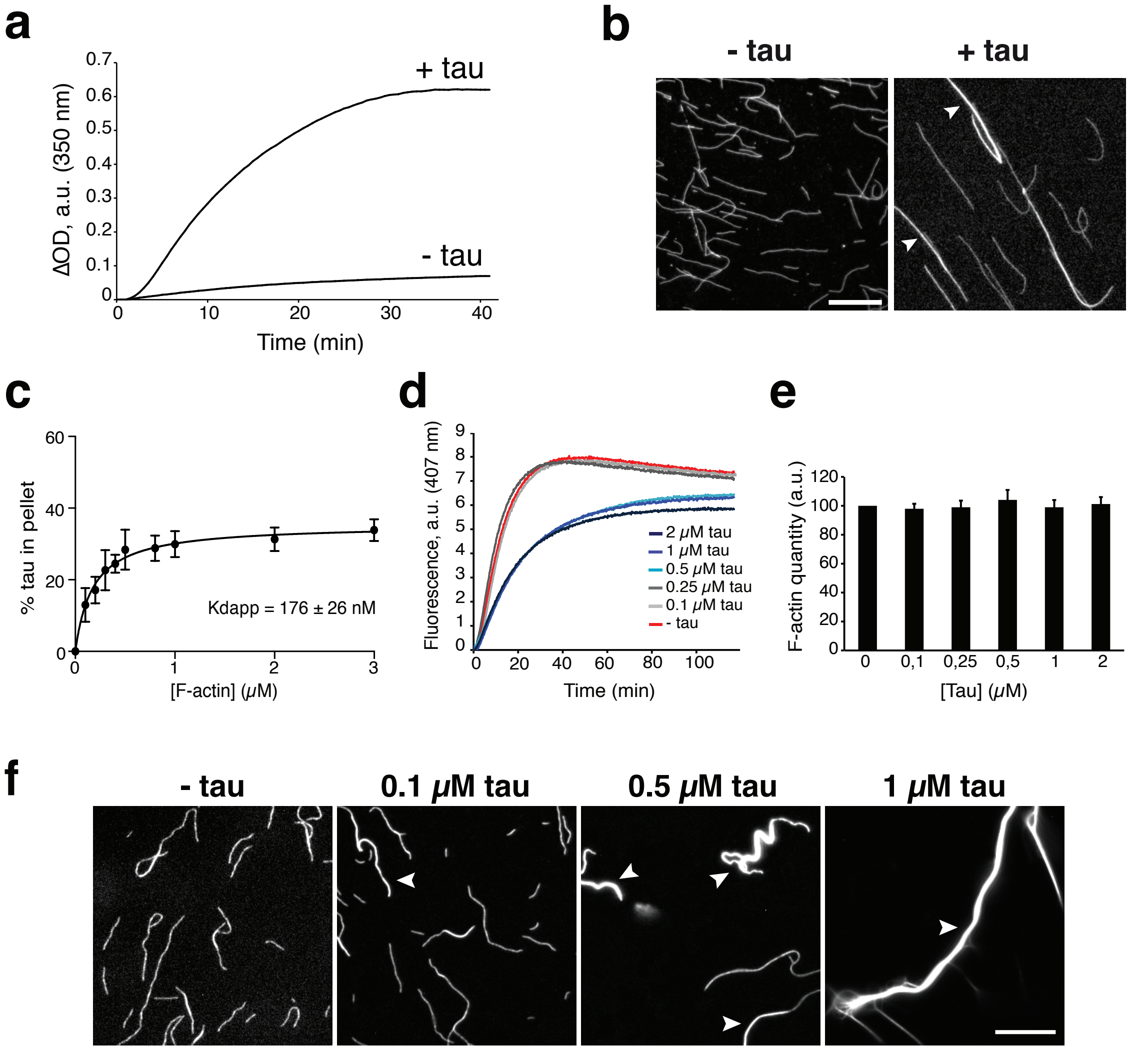

Supplementary Figure S1. Effects of tau on microtubule and actin assembly.
(a) Turbidity measurements of tubulin polymerization at 350 nm. The addition of tau (1 μM) to tubulin (20 μM) promotes microtubule self-assembly. (b) Fluorescence light microscopy of microtubules polymerized from 60 μM fluorescent tubulin alone (left) or 20 μM fluorescent tubulin with 1 μM tau (right). Samples were fixed and centrifuged onto coverslips before observation. Arrowheads indicate microtubule bundles. Scale bar, 10 μm. (c) Determination of the apparent binding affinity of tau for F-actin in BRB80-K buffer. Increasing concentrations of F-actin were incubated with 0.5 μM tau. The percentage of bound tau was plotted against F-actin concentration and fitted with a hyperbolic function. The Kdapp value was 176 ± 26 nM (mean ± SD, five independent experiments). (d) Time course of actin polymerization (4 μM actin monomers) in the presence of increasing tau concentrations (0.1, 0.25, 0.5, 1 and 2 μM) monitored by pyrene-actin fluorescence. a.u., arbitrary fluorescence units. Tau does not affect actin polymerization, except at concentrations above 1 μM that induce a decrease in the extent of actin assembly. However, quantification of actin polymerization in same conditions by co-sedimentation (e) reveals similar quantities of pelleted F-actin. Quenching of actin-pyrene fluorescence upon tau binding might explain the decrease in fluorescence observed at the highest tau concentrations. (e) Quantification of actin polymerization by high-speed sedimentation assay. Tau was incubated for 5 min with actin under the same conditions as in (d) before being centrifuged. The amount of polymerized F-actin is plotted as a function of tau concentration. Data represent the mean ± SD (three independent experiments). (f) Tau-induced F-actin bundles observed by fluorescence light microscopy. Arrowheads indicate F-actin bundles. Scale bar, 10 μm.

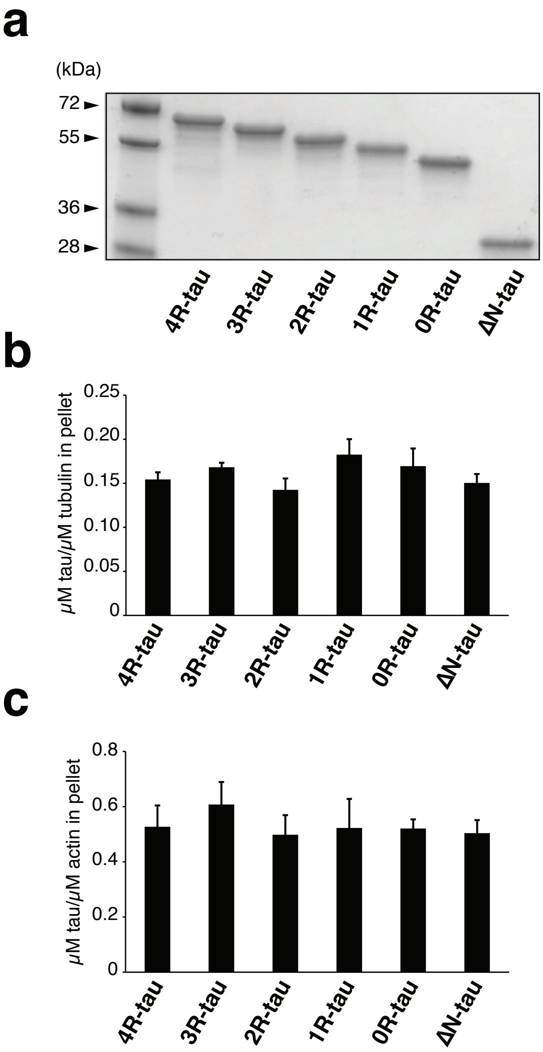

Supplementary Figure S2. Relative binding of tau deleted forms to microtubules and to actin filaments.

(a) Coomassie-stained SDS-PAGE gel showing the different tau proteins used in this study. About 0.7 µg of each protein was loaded. (b) Quantification of the amount of tau proteins bound to microtubules in similar ratio and buffer as the ones used in TIRF microscopy assays (Fig. 3). Tubulin and tau proteins were incubated in BRB80-K buffer supplemented with 1 mM GTP in a 7:1 molar ratio (60 µM tubulin for 8.4 µM tau) for 30 minutes at 32°C. Samples were centrifuged on a 60% sucrose cushion for 30 minutes at 230,000 x g and the pellet and supernatant were separated and quantified after SDS-PAGE. Data represent the mean ± SD (three independent experiments). (c) Quantification of the amount of tau proteins bound to actin filaments in similar ratio and buffer conditions used in TIRF microscopy assays (Fig. 3). Actin (1 µM) was incubated in BRB80-K buffer supplemented with 1 mM GTP for 30 minutes at 32°C with 1.75 µM 4R-tau or ΔN-tau, 5.25 µM 3R tau, 7 µM 2R- or 1R-tau and 7.5 µM 0R-tau. Samples were centrifuged for 30 minutes at 100,000 x g and the supernatants and pellets were separated and quantified after SDS-PAGE. Data represent the mean ± SD (three independent experiments).

Supplementary Methods:

Microtubule self-assembly. For tubulin polymerization, 20 µM tubulin was incubated with or without 1 µM 4R-tau at 36°C in BRB80-K buffer supplemented with 1 mM GTP. Microtubule self-assembly was monitored at 350 nm using an UVIKON XS spectrophotometer. Fluorescent microtubules were polymerized in the same conditions in the presence of tubulin containing 10% of ATTO-565 labeled tubulin. One ml of BRB80 buffer containing 0.5% glutaraldehyde and 25% (w/v) sucrose was added to 20 µl of reactions and the samples were sedimented onto a BRB80-10% glycerol onto coverslips, fixed in methanol and observed by fluorescence microscopy as described in the experimental procedure.

Tau interaction with F-actin in BRB80-K buffer. To determine tau affinity for F-actin in BRB80-K buffer, phalloidin-stabilized F-actin was prepared from 30 µM G-actin incubated for 1h at RT with 30 µM phalloidin in AP (Actin Polymerization) buffer. Increasing amounts of phalloidin-stabilized F-actin were incubated with 0.5 µM 4R-tau in BRB80-K buffer for 30 min at RT. Samples were centrifuged at 100,000 x *g* for 15 min and the amount of 4R-tau bound to F-actin in the pellets was quantified as described in Methods.

Actin polymerization and bundling. Increasing amounts of 4R-tau was added to 4 µM G-actin containing 10% of pyrene-labeled G-actin in AP buffer and pyrene fluorescence was measured at 407 nm at RT using a Safas Xenius XC spectrophotometer. To quantify the amount of F-actin polymerized with tau, similar reactions were incubated for 5 min at RT before being centrifuged for 10 min at 100,000 x *g*. In non-polymerizing conditions (AP buffer without KCl, imidazole, MgCl2 and EGTA), negligible amount of actin was found in the pellet. To visualize tau-induced F-actin bundles, 4 µM G-actin was polymerized with

increasing amounts of 4R-tau (0, 0.1, 0.5 and 1 µM) and 4 µM phalloidin-ATTO-565 during 30 min at RT before observation under a fluorescence microscope.

Supplementary Movie Legends:

Supplementary Movie S1. Tau promotes the bundling of actin filaments. TIRF movies of actin (0.4 µM) polymerizing in the absence (left) or in the presence of 0.1 µM tau (right). The time (min:s) represents the elapsed time from the start of the acquisition. Scale bar, 10 µm.

Supplementary Movie S2. Tubulin and actin co-assembly in the absence or in the presence of fascin. Dual-color TIRF movies of tubulin (green) and actin (red) co-assembling in the absence (left) or in the presence (right) of fascin. The time (min:s) represents the elapsed time from the start of the acquisition. Scale bar, 10 µm.

Supplementary Movie S3. Tau co-organizes growing microtubule and actin networks. Dual-color TIRF movie of tubulin (green) and actin (red) co-polymerizing in the presence of tau. The time (min:s) represents the elapsed time from the start of the acquisition. Scale bar, 10 µm.

Supplementary Movie S4. Detailed views of microtubule/actin coupling in the presence of tau. Dual-color TIRF movies illustrating tau-induced microtubule/actin interactions. Part A shows guided polymerization of F-actin along microtubule tracks. Part B shows the progressive straightening of F-actin by the end of a growing microtubule. Part C shows a microtubule elongating along F-actin bundles. The time (min:s) represents the elapsed time from the start of the events. Scale bar, 5 µm.

Supplementary Movie S5. The projection domain of tau is not necessary for its microtubule/actin cross-linking activity. Dual-color TIRF movies of tubulin (green) and actin (red) co-assembling in the presence of 4R-tau (left) or ΔN-tau (right). The time (min:s) represents the elapsed time from the start of the acquisition. Scale bar, 10 μm.

Supplementary Movie S6. The tubulin-binding sites of tau are required to link microtubules and actin filaments. Dual-color TIRF movies of tubulin (green) and actin (red) co-polymerizing with 3R-, 2R-, 1R- or 0R-tau. The time (min:s) represents the elapsed time from the start of the acquisition. Scale bar, 10 μm.
